# The Carbon Footprint of Diets with Different Exclusions of Animal-Derived Products: Exploratory Polish Study

**DOI:** 10.3390/nu17081377

**Published:** 2025-04-19

**Authors:** Anna Choręziak, Piotr Rzymski

**Affiliations:** Department of Environmental Medicine, Poznan University of Medical Sciences, 60-806 Poznań, Poland; anna.choreziak@gmail.com

**Keywords:** plant-based diets, animal products, environment, sustainability, climate change

## Abstract

**Background/Objectives:** Analyzing the carbon footprint of diets in various populations is important as it can help identify more sustainable food choices that reduce the overall impact of human activities on ongoing warming of the global climate. This pilot exploratory study analyzed the carbon footprint (measured in kg of CO_2_ equivalent, eq.) using food diaries collected from Polish individuals with varying levels of animal-derived product exclusion in their diets. **Methods:** The study employed a food diary method, where participants from four dietary groups (vegan, vegetarian, fish-eater, and meat-eater) recorded all meals and beverages consumed over a 7-day period, including portion sizes and packaging details. These diaries were then analyzed to assess dietary adherence and calculate carbon footprints, utilizing standardized CO_2_ equivalent emission data from publicly available databases. **Results:** The analysis revealed a decreasing trend in the carbon footprint corresponding to the degree of elimination of animal-derived products from the diet (R^2^ = 0.96, *p* = 0.0217). The mean daily footprint in the vegan group was 1.38 kg CO_2_ eq., which was significantly lower than in the vegetarian (2.45), fish-eater (2.72), and meat-eater groups (3.62). For each 1000 kcal, the meat-eater diet generated 39.7, 58.3, and 93.9% more CO_2_ eq. than in the case of fish-eaters, vegetarians, and vegans, respectively. Over a week, a group of 10 vegans had a total carbon footprint lower than vegetarians, fish-eaters, and meat-eaters by 42.9, 52.2, and 61.8%, respectively. Hard and mozzarella cheese had the highest contribution to the carbon footprint in vegetarians, fish, and seafood in fish-eaters, and poultry, pork, and beef had the highest contribution in meat-eaters. **Conclusions:** Dietary carbon footprints vary considerably by dietary pattern, with lower consumption of animal-derived products associated with lower emissions. Additionally, identifying specific high-impact food items within each diet may inform strategies for reducing environmental impact across various eating patterns.

## 1. Introduction

The vast majority of scientific evidence unequivocally indicates that the main cause of observed global warming is the emission of greenhouse gases, predominantly CO_2_, originating from human activity [1]. Ongoing climate change already has multidimensional and adverse economic, social, health, and environmental effects, which are predicted to become more severe without mitigation measures and adaptation strategies [2,3,4]. A global food emissions database, EDGAR-FOOD, estimated greenhouse gas emissions in the 1990–2015 period and estimated that one-third of these emissions are related to the global food system, from production to consumption [5]. The most significant of these contributions (over 70%) were related to agricultural and land use and change activities [5]. Moreover, animal agriculture itself was demonstrated to be responsible for up to 20% of global CO_2_ emissions [6]. A meta-analysis of the environmental impacts of over 38,000 agricultural farms producing various food goods worldwide demonstrated that beef, lamb, mutton, and dairy had the highest carbon footprint [7].

All of these findings indicate that individual choices may indeed have a varying impact on greenhouse emissions and, by extension, on climate and further climate changes. In other words, certain dietary modifications may be perceived as measures to mitigate or minimize the adverse impacts of human activity on the environment [8]. As recently assessed with a global economic land model, substituting 50% of globally consumed beef, chicken, milk, and pork with plant-based alternatives would lead to substantial environmental benefits, including a decline of greenhouse gas emissions by over 30% in 2050 compared to 2020 [8]. Environmental concerns have also been addressed in the Special Report on Climate Change and Land, which was prepared by the Intergovernmental Panel on Climate Change [9]. It recognizes with high confidence that the adoption of diets higher in plant-based foods, such as vegetables, fruits, whole grains, legumes, nuts, and seeds, and lower in animal-sourced foods, fats, and sugar, has significant potential for mitigation [9].

Therefore, assessing the environmental impacts of diets that differ in excluding and including particular food products is valuable. Since diets may vary significantly between individuals and regions, food diaries allow for the personalization of carbon footprint estimates because they consider specific food choices, quantities, and meal patterns [10], which is essential for understanding the impact of an individual’s diet on the environment.

Although such studies were conducted in various populations [11,12,13], data on the environmental impacts of the diets of Polish consumers are scarce. So far, this has been addressed only by one recent study comparing 24 individuals who presented plant-based and omnivorous diets based on 3-day dietary records [14]. It is necessary to conduct further research in this regard that would include different diet types, including pescatarians (fish-eaters), and tracking the dietary record for at least a week. Such data are essential for appropriate communication with the public in Poland, who may remain unconvinced about the concept of limiting environmental impacts through dietary changes unless locally relevant data are generated. Having such data is important since survey studies show that although more than half of Poles consider climate change to be a concern, and they undertake some measures to lessen their effect on carbon footprint, these measures rarely include changes in the diet [15,16].

Therefore, the present exploratory study aimed to assess the carbon footprint of Polish adults who differ in their exclusion of animal-derived products according to their weekly food diaries. To this end, individuals representing four different dietary patterns were enrolled: vegans, vegetarians, fish-eaters (pescatarians), and meat-eaters (omnivores), while their diet was tracked for one week. The results of such a study can help tailor sustainability efforts and promote public awareness of the environmental impacts of diets in Poland.

## 2. Materials and Methods

### 2.1. Study Participants

The recruitment process for study participants began by defining the eligibility criteria, which included Polish nationality, age over 18 years, and representing one of the dietary patterns: vegan, vegetarian, fish-eater, or meat-eater. There were no specific exclusion criteria regarding participants’ health status, presence of chronic diseases, or supplementation use. Participants were not asked to report any existing medical conditions or supplement intake, as the aim of the study was to reflect real-life dietary behaviors without imposing additional restrictions. The study, conducted at the Department of Environmental Medicine of Poznan University of Medical Sciences, took place in October–December 2023, and participants were recruited on a voluntary basis through social media platforms. Potential participants were informed about the study’s purpose and procedures. They were free to withdraw from the study at any stage. All study participants gave consent to participate and were not paid or rewarded in any way. Each participant was asked to complete a weekly food diary using tables. One dietary form was completed per participant, covering the entire 7-day period. The completed food diaries were analyzed by the author of the study (A.C.), who is a qualified nutrition specialist. The diary included information about the quantity and composition of meals and drinks consumed during the day. Since the study was not a medical experiment, approval from the Bioethical Committee was waived according to Polish law.

### 2.2. Dietary Assessment

The form used for the dietary assessment was specifically designed for this study (Supplementary Material). Participants were asked to record all foods and beverages consumed, including portion sizes (e.g., tablespoon or teaspoon; glass with volume in mL; slice; piece) and weights (participants were encouraged to use kitchen scales when possible and online resources, e.g., www.ilewazy.pl, accessed on 1 January 2024). Additionally, participants were asked to note the packaging type (e.g., plastic packaging, paper bag, reusable bag, glass bottle, aluminum can), although this information was not included in the analysis. The table included meals such as breakfast, second breakfast, lunch, afternoon snack, dinner, and any snacks consumed throughout the day. Participants were instructed to record only those meals they had consumed. A correctly completed example for one day was also provided with instructions for reference (Supplementary Material). Participants could contact the research team by e-mail or phone if they had any questions, using the contact information provided along with the food diary instructions.

The food diaries that were obtained were analyzed in terms of the type of meals and drinks consumed and their quantity. The diet energy value was estimated using the Dietetyk Pro (2023) software (AuraGroup, Wrocław, Poland). Adherence to the declared diet was assessed based on the actual contents of the food diaries, not solely on participants’ self-reported dietary patterns. Each participant’s weekly intake was cross-verified against accepted definitions of dietary patterns. A participant was confirmed as: (1) vegan if the food diary contained no animal-derived products, including dairy, eggs, fish, meat, or honey; (2) vegetarian if the diary included dairy products, eggs, or honey, but excluded fish, meat, and poultry; (3) fish-eater (pescatarian) if the diary included fish along with dairy, eggs, and/or honey, but excluded meat and poultry; (4) meat-eater (omnivore) if any form of meat or poultry was consumed, along with other animal products.

A total of 40 weekly food dairies, collected from 10 individuals representing each diet pattern (vegan, vegetarian, fish-eater, meat-eater), were analyzed. The age and sex distribution varied across dietary groups. In the meat-eaters group (*n* = 10), the mean age was 44 years (range: 24–66), with six female and four male participants. The fish-eaters group (*n* = 10) had a mean age of 34 years (range: 22–42), including five females and five males. The vegetarian group (*n* = 10) had a mean age of 29 years (range: 23–39), including eight female and two male participants. The vegan group (*n* = 10) had a mean age of 31 years (range: 24–37), with 10 female participants and no males.

### 2.3. Carbon Footprint Assessment

The environmental footprint of the study participants’ diets was assessed using publicly available average data on the CO_2_ equivalent (CO_2_ eq.) emissions of food products. The CO_2_ eq. metric takes into account the emissions of various greenhouse gases converted to the equivalent amount of CO_2_ with the same global warming potential, which allows for a direct comparison of the climate impacts of different products [17]. For this purpose, the following databases were used: CarbonCloud ClimateHub (https://apps.carboncloud.com/climatehub, accessed on 1 January 2024), FoodFootprint (foodfootprint.nl, accessed on 1 January 2024), and The Big Climate Database (https://denstoreklimadatabase.dk/en, accessed on 1 January 2024). Based on the quantities of products consumed by the participants, the carbon footprint of each diet, expressed as kg CO_2_ equivalent, was calculated. In addition, the carbon footprint of each participant’s diet was also standardized based on its energetic value, in order to obtain emissions of CO_2_ eq. values per each 1000 kcal consumed.

### 2.4. Statistical Analyses

The results were analyzed with PQStat v.1.8.2 (PQStat Software, Poznan, Poland). The data distribution was analyzed using the Kolmogorov–Smirnov test, and parametric methods were applied due to adherence to the Gaussian distribution. The difference in carbon footprint, age, and daily energy intake between dietary groups was analyzed by one-way analysis of variance (ANOVA) with a post-hoc Tamhane’s T2 test. Sex distribution in particular groups was compared with Pearson’s chi-square method. The trend in carbon footprint depending on the level of elimination of animal-derived products from the diet (vegans > vegetarians > fish-eaters > meat-eaters) was analyzed with a linear regression function and the coefficient of determination (R^2^). The association between daily energy intake and carbon footprint was evaluated using Spearman’s rank correlation coefficient (Rs). A *p*-value of less than 0.05 was considered statistically significant in all analyses.

## 3. Results

The distributions of participants’ age, sex, and daily energy intake are summarized in Table 1. The age and daily energy intake did not differ between the groups (*p* = 0.07). The groups varied in sex distribution (*χ*^2^ = 70.2, *p* < 0.0001) and, except for fish-eaters, were dominated by females.

**Figure 1:**
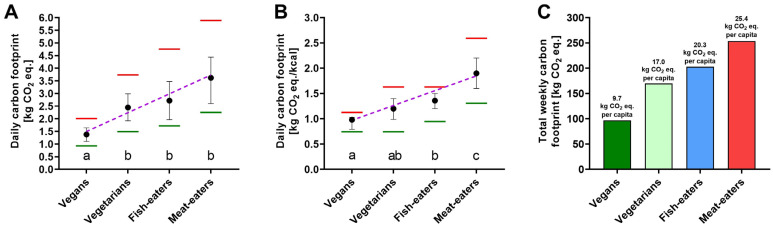
The carbon footprint of a particular diet group (n = 10) presented as (**A**) total daily CO_2_ eq. and (**B**) daily CO_2_ eq. standardized over energy intake. Points represent mean, whiskers represent a 95% confidence interval, green and red lines represent minimal and maximum value, respectively, and the dotted violet line represents the best-fit line between the level of elimination of animal-derived products and carbon footprint. Different letters denote statistically significant differences (*p* < 0.05) between groups in post-hoc tests following ANOVA; the same letters indicate no differences between compared groups. (**C**) Total weekly carbon footprint of each group consisting of 10 individuals with mean per capita values given above the bars.

The analyzed dietary groups revealed differences in carbon footprint (*p* = 0.000149, ANOVA). A significant linear trend existed between the level of elimination of animal-derived products and carbon footprint (*y* = −0.6990x + 4.290, *R*^2^ = 0.96, *p* = 0.0217). The lowest mean daily carbon footprint was observed in vegans, for whom it was lower by 43.7, 49.3, and 61.9% compared to vegetarians, fish-eaters, and meat-eaters, respectively (Figure 1). Moreover, the maximum value of the daily carbon footprint observed in the vegan group was lower than the minimal value of this parameter in fish- and meat-eaters (Figure 1A). Similarly, the maximum carbon footprint per 1000 kcal was found for meat-eaters, for whom the mean value of this parameter was 39.7, 58.3, and 93.9% higher than in the case of fish-eaters, vegetarians, and vegans, respectively (Figure 1B). The vegan group also revealed the lowest total weekly carbon footprint, amounting to 97 kg CO_2_ eq., lower than for vegetarians, fish-eaters, and meat-eaters by 73, 106, and 181 kg CO_2_ eq., respectively (Figure 1C).

Energy intake (Table 1) correlated with CO_2_ eq. emissions (Figure 1B) in all analyzed groups: meat-eaters (Rs = 0.64, *p* = 0.04), fish-eaters (Rs = 0.92, *p* = 0.0002), vegetarians (Rs = 0.61, *p* = 0.04), and vegans (Rs = 0.61, *p* = 0.03). Table 2 summarizes the percentage of contribution of particular food products to the carbon footprint in different dietary groups. Among the products consumed by the vegetarian group, hard and mozzarella cheeses revealed the highest contribution to the carbon footprint. In turn, fish and seafood consumption had the greatest contribution in this regard among fish-eaters. In the case of meat-eaters, over 40% of the carbon footprint was, on average, originating from poultry, pork, and beef consumption, while in selected individuals, it exceeded 70% (Table 2).

## 4. Discussion

The present exploratory study, conducted on a group of Polish adults, adds to the mounting evidence that eliminating or reducing animal-derived products from the diet can represent a climate change mitigation strategy that can be undertaken individually. This is highly important as it may inspire climate action instead of climate despair (also referred to as “eco-anxiety”) that emerges from the conviction that one’s decisions are meaningless in the face of such a vast and complex threat as global warming [18,19,20]. Analyzing the carbon footprint of diets can empower consumers to make more environmentally conscious decisions. With increased awareness, people can choose diets that align with their values regarding sustainability, such as opting for plant-based meals or reducing meat consumption [21].

Generally, as shown using trend analysis, the pressure of diet on the climate decreased with the level of reduction of products of animal origin. Similar results were obtained in research conducted in other populations. For example, the US study showed exactly the same trend as the present investigation, with a vegan diet having the lowest carbon footprint, followed by vegetarian, pescatarian, and omnivore diets. In addition, the paleolithic and ketogenic diets were demonstrated to be even more climate unfriendly, with the latter having an over 4-fold and nearly 2-fold higher carbon footprint compared to vegan and omnivore diets, respectively [22]. Another study showed that vegan and vegetarian diets based on traditional Turkish cuisine had a 48 and 21% lower carbon footprint compared to the omnivore diet [23]. The research, which applied a Life Cycle Assessment to evaluate the carbon footprint of different diet scenarios in Denmark, also clearly showed that vegan and vegetarian diets had the best profile, and switching to them would decrease greenhouse emissions by 0.7 and 0.2 tonnes CO_2_ eq. per person per year [24]. While these environmental benefits are increasingly well-established, there remains a lack of data contextualized to specific populations. The importance of the present study lies in its focus on the Polish context, where dietary patterns, cultural preferences, and public awareness around sustainability may differ significantly from Western settings more commonly represented in the literature.

The investigation conducted in Italy indicated that vegan and vegetarian meals served in hospitals were not only more climate-friendly as measured by carbon footprint but also more in line with the Italian dietary reference values than omnivorous meals [25]. These findings were consistent in the Polish setting as the lowest mean daily carbon footprint was demonstrated for vegan groups, with a maximum value for this parameter that was lower than the minimum level found for meat-eaters. However, one should note that in all studied groups, energy intake correlated positively with carbon footprint. Therefore, there might be some significant differences in environmental impacts between low- and high-caloric plant-based diets, though even the latter will be more sustainable than omnivorous eating patterns. In general, the carbon footprint decreased with the level of elimination of animal products from the diet, which was also seen when the CO_2_ eq. values were standardized over energy intake. Furthermore, we show that the weekly contribution of the analyzed group of 10 vegans to carbon emissions was decreased by 181 kg CO_2_ eq. compared to meat-eaters. Using this data, and considering the hypothetical scenarios under which the entire Polish adult population (30.8 million in 2022 [26]) would be represented by meat-eaters or vegans, the yearly carbon footprint of their diet would amount to 40.7 and 15.5 million metric tones of CO_2_ eq., respectively The difference of 25.2 million metric tons of CO_2_ eq. between these diets would equal 38% and 84.9% of total yearly CO_2_ emissions from the transport and industry sector, as reported by the International Energy Agency in 2021 for Poland [27]. Although these figures shall be treated as approximations, they clearly show that a plant-based diet can substantially contribute to decarbonization.

Although plant-based diets, such as a vegan diet, could be seen as a solution to various issues associated with meat production and consumption [28,29], including its impact on climate change, and could provide some health benefits [30], it is likely that a vast majority of consumers are unwilling to change their dietary patterns radically and entirely eliminate animal-derived foods [31]. Some studies argue that the potential solution to this challenge lies in introducing cultivated meat obtained from animal cells cultured in vitro [29]. This can potentially offer several environmental benefits, including reductions in energy consumption, land use, water withdrawal, and greenhouse gas emissions, while often described in the literature as ethically preferable to conventional meat due to animal welfare, and providing food safety benefits, reducing some of the health threats related to its production, and not requiring consumers to exclude it from their diet [32,33]. However, public acceptance of such products is uncertain, particularly in more conservative populations. As recently shown, over half of the surveyed Polish adults declared their willingness to buy cultivated meat when it becomes available, with meat-eaters being one of the major groups interested in accepting such a product [34]. Furthermore, as recently argued, it remains to be shown whether meat produced in such a manner can truly be the nutritional equivalent of its conventional counterpart [35]. On the other hand, it is worth noting that according to a study that employed Google Trends to analyze regional interest in different dietary patterns, veganism was the most frequently searched diet in Poland [36]. Therefore, the result of the present study may provide further arguments in favor of the transition to diets that exclude animal products, in addition to the ethical motivation, which is the most frequent reason for selecting veganism [37]. This may be especially important for young Poles since this group reveals the highest preference for excluding animal products from their diet [38].

The present study shows that potential environmental benefits in terms of minimizing the carbon footprint could be achieved by reducing the amount of animal-derived products without shifting to another diet type. For example, a decrease in vegetarians’ cheese consumption could further lower their dietary carbon footprint. This could be achieved by increasing the consumption of other products in their diet, e.g., vegetables, and/or introducing cheese alternatives made from legumes, rice, almonds, and nutritional yeast, the market for which is currently growing. Similarly, the reduction in carbon footprint in a group of meat-eaters could be achieved by a more balanced consumption of poultry, pork, and beef. This could have simultaneous health benefits since the intake of meat in Poland, especially red and processed meat, remains high and frequent, exceeding the recommendations and potentially increasing the risk of selected chronic diseases [39]. Education efforts, preferably integrating health and environmental goals of reducing meat consumption, must be pursued to achieve this goal.

Certain study limitations must be stressed. Firstly, although our exploratory research filled the gap in analyzing the carbon footprint of diets in the Polish setting, included four different dietary patterns, and employed food diaries, it should be noted that each analyzed group was small. Therefore, the results should not be extrapolated to the entire population and may have limited generalizability, especially regarding the differences between meat-eaters, fish-eaters, and vegetarians, which, contrary to the vegan group, were not statistically significant in terms of carbon footprint. Instead, the results provide a snapshot of differences in climate impacts of different diets practiced in Poland and motivations for further research. While the observed effect sizes were notable, the current sample size does not allow for robust conclusions regarding differences between dietary groups, especially given the heterogeneity in sex distribution. As such, the findings should be interpreted with caution and viewed primarily as a foundation for future research. Second, the study was conducted during the autumn–winter season in Poland; therefore, it did not account for seasonal changes in the availability of selected foodstuffs, especially those of plant origin, such as asparagus and pumpkin. Further investigations would be required to explore how this aspect could affect the carbon footprint of a particular diet. Third, the climate impacts of particular foods consumed by the individuals being studied were calculated based on generalized data gathered by publicly available databases on the carbon footprint of foodstuffs, while environmental impacts may differ not only due to the type of product but also the process of its production (e.g., organic vs. non-organic) and transport type (though this aspect would significantly affect only the minority of foodstuffs in Poland that are transported by air) [40]. Fourth, we acknowledge that the female-to-male ratio in our study is unbalanced. However, the study sample reflects the characteristics of the population willing to participate and is consistent with participation trends observed in similar research. This gender imbalance may be partly explained by the fact that women are more likely to adopt vegetarian diets and are generally more willing to participate in research studies and surveys [41,42,43,44]. Despite the fact that a longer monitoring period for consumed products would provide a broader picture, a one-week food diary was used for practical reasons, primarily due to participant burden and the feasibility of maintaining accurate dietary records over an extended period. Research indicates that a one-week dietary assessment can provide reliable information about an individual’s typical intake, particularly if the week is representative of their usual eating patterns [45,46]. Future extensions of this research may involve recruiting a larger, demographically balanced sample to improve statistical power and allow subgroup analyses. Moreover, follow-up studies may consider longitudinal designs or interventions to assess not only the environmental footprint but also changes in dietary behavior and attitudes toward sustainable eating in response to education or policy shifts.

## 5. Conclusions

The findings of this exploratory study in a group of Poles underscore the significant impact of dietary choices on carbon emissions, emphasizing the importance of shifting towards more sustainable eating habits to mitigate global climate change. The clear trend that we observed—wherein the carbon footprint diminishes as the consumption of animal-derived products decreases—highlights the potential of plant-based diets, particularly veganism, in reducing individual contributions to greenhouse gas emissions. The stark differences in carbon footprints between vegans and other dietary groups, with vegans producing substantially lower emissions, support the growing body of evidence that advocates for reduced reliance on animal products as a critical component of climate action.

Moreover, this research not only affirms the environmental benefits of veganism but also suggests that even partial reductions in animal product consumption, such as those seen in vegetarian, fish-based, and meat-including diets, can lead to meaningful decreases in carbon emissions. The identification of specific foods with high climate impact within each dietary group provides actionable insights for individuals and policymakers alike, pointing to targeted areas where dietary modifications can be most effective. Ultimately, while a complete shift to veganism offers the greatest reduction in carbon footprint, the study also encourages more modest dietary adjustments as a viable and impactful step towards greater sustainability.

However, these conclusions must be interpreted with caution due to the study’s limited sample size and demographic variability. As a pilot investigation, the findings are intended to offer initial insights and generate hypotheses for future, more comprehensive research on diet-related climate impacts in Poland, which is highly encouraged.

## Figures and Tables

**Table 1:** The distribution of participants according to age, sex, and daily energy intake.

	Daily Energy Intake (Mean ± SD), kcal	Age (Mean ± SD), Years min/max	Female/Male, %
Meat-eaters (*n* = 10)	1902 ± 544	44 ± 17 24/66	60/40
Fish-eaters (*n* = 10)	1984 ± 604	34 ± 7 22/42	50/50
Vegetarians (*n* = 10)	2009 ± 337	29 ± 5 23/39	80/20
Vegans (*n* = 10)	1509 ± 277	31 ± 5 24/37	100/0

**Table 2:** The mean (min–max) percentage of contribution of particular food products to carbon footprint (CO_2_ eq.) in diets that include varying levels of animal-derived products.

	Vegans	Vegetarians	Fish-Eaters	Meat-Eaters
Hard and semi-hard cheese	-	20 (6–29)	11 (0–26)	2 (0–4)
Cow milk and fermented milk drink	-	4 (0–14)	6 (0–21)	8 (1–29)
Cottage cheese	-	6 (0–8)	3 (1–9)	3 (0–12)
Eggs	-	2 (0–8)	4 (0–11)	3 (1–6)
Fish	-	-	18 (2–29)	3 (0–9)
Meat (poultry, pork, beef)	-	-	-	44 (29–71)
Grains	8 (2–15)	6 (0–8)	4 (0–9)	2 (1–4)
Legumes	6 (3–8)	2 (0–6)	1 (0–5)	1 (0–1)
Nuts	1 (0–4)	1 (0–4)	1 (0–2)	1 (0–2)
Bread	6 (0–8)	5 (3–13)	5 (2–5)	3 (1–5)
Fruits	3 (0–7)	2 (0–6)	3 (0–8)	2 (0–3)
Vegetables	14 (8–17)	7 (3–9)	6 (1–13)	4 (2–5)
Tea	1 (0–2)	1 (0–1)	1 (0–2)	1 (0–2)
Coffee	5 (0–8)	2 (1–3)	2 (0–8)	1 (0–2)
Other drinks	15 (3–37)	13 (0–21)	10 (0–27)	5 (0–18)

## Data Availability

The data supporting the conclusions of this article will be made available by the authors upon reasonable request.

## Supplementary Materials

The following supporting information can be downloaded at: https://www.mdpi.com/article/10.3390/nu17081377/s1 (Translated dietary form and translated instructions for participants).
