# Supplementary material for: The Carbon Footprint of Diets with Different Exclusions of Animal-Derived Products: Exploratory Polish Study
Source: Nutrients. 2025 Apr 19;17(8):1377. doi: 10.3390/nu17081377 (PMC12030518; doi:10.3390/nu17081377)
Supplement: Supplementary file 1 [file nutrients-17-01377-s001.zip › 7-Day_Food_Diary INSTRUCTION.pdf]

7-Day Food Diary

Instructions:
Please record all foods and beverages consumed, including household measures (e.g., tablespoon, glass, slice, piece) and weight in grams (a kitchen scale or the website ilewazy.pl may be useful). Also, note the type of packaging the product was purchased in (e.g., plastic packaging, paper bag, reusable bag, glass bottle, aluminum can).

Try to be as specific as possible, including brand names, preparation methods (e.g., boiled, fried, raw), and any additions or condiments used (e.g., sugar in tea, butter on bread).
Please complete the diary on the same day as the consumption to avoid forgetting details.

Example Day

Meal	Food Items	Household Measure	Weight (g/mL)	Packaging Type
Breakfast	Apple	piece	120 g	own bag
	Bread- rye	slice	80 g	paper
	Butter	0.5 teaspoon	2.5 g	plastic
	Cheese (yellow)	2 slices	60 g	plastic
	Grapefruit juice	glass	200 mL	glass
Second Breakfast	Energy drink	200ml	200 mL	can
	Graham roll	piece	90 g	paper
	Peanut butter	tablespoon	40 g	glass
	Jam- mixed fruit	tablespoon	40 g	glass
Lunch	Potatoes	2 pieces	120 g	own bag
	Cucumber	1	70 g	own bag
	Dill	tablespoon	10 g	foil

	Natural yogurt	3 tablespoons	60 g	plastic
	Eggs, fried	2	120 g	carton
	Rapeseed oil	teaspoon	5 g	glass
Afternoon Snack	Hummus	package	150 g	plastic
	Carrot	piece	45 g	own bag
	Bell pepper	0.5 piece	115 g	foil
	Bread rye	slice	80 g	foil
Dinner	Pizza	3 portions	600 g	carton
	Cola	glass	200 mL	can
	Rucola	handful	20 g	plastic
	Tomato	piece	120 g	own bag
	Garlic	clove	5 g	own bag
	Olive oil	teaspoon	5 g	glass
Snack	Chewing gum	sheet	5 g	plastic
	Mineral water	bottle	1.5 L	plastic
	Chips	packet	40 g	plastic
