# Supplementary material for: The Carbon Footprint of Diets with Different Exclusions of Animal-Derived Products: Exploratory Polish Study
Source: Nutrients. 2025 Apr 19;17(8):1377. doi: 10.3390/nu17081377 (PMC12030518; doi:10.3390/nu17081377)
Supplement: Supplementary file 1 [file nutrients-17-01377-s001.zip › 7-Day Food Diary.pdf]

7-Day Food Diary

Monday

Meal	Food Items	Household Measure	Weight (g/mL)	Packaging Type
Breakfast				
Second Breakfast				
Lunch				
Afternoon Snack				
Dinner				
Snack				

Tuesday

Meal	Food Items	Household Measure	Weight (g/mL)	Packaging Type
Breakfast				
Second Breakfast				
Lunch				
Afternoon Snack				
Dinner				
Snack				

Wednesday

Meal	Food Items	Household Measure	Weight (g/mL)	Packaging Type
Breakfast				
Second Breakfast				
Lunch				
Afternoon Snack				
Dinner				
Snack				

Thursday

Meal	Food Items	Household Measure	Weight (g/mL)	Packaging Type
Breakfast				
Second Breakfast				
Lunch				
Afternoon Snack				
Dinner				
Snack				

Friday

Meal	Food Items	Household Measure	Weight (g/mL)	Packaging Type
Breakfast				
Second Breakfast				
Lunch				
Afternoon Snack				
Dinner				
Snack				

Saturday

Meal	Food Items	Household Measure	Weight (g/mL)	Packaging Type
Breakfast				
Second Breakfast				
Lunch				
Afternoon Snack				
Dinner				
Snack				

Sunday

Meal	Food Items	Household Measure	Weight (g/mL)	Packaging Type
Breakfast				
Second Breakfast				
Lunch				
Afternoon Snack				
Dinner				
Snack				
